# The enigma of glutamine synthetase and b-catenin expression in hepatocellular adenoma in familial adenomatous polyposis coli

**DOI:** 10.1007/s00428-024-03772-1

**Published:** 2024-03-08

**Authors:** Annette S. H. Gouw, Christine Sempoux, Paulette Bioulac-Sage

**Affiliations:** 1 Department of Pathology and Medical Biology, University Medical Center Groningen, Groningen, The Netherlands; 2 Service of Clinical Pathology, Institute of Pathology, Lausanne University Hospital and University of Lausanne, Lausanne, Switzerland; 3 University of Bordeaux, INSERM, BRIC, U1312 Bordeaux, France

**Keywords:** Hepatocellular adenoma, Familial adenomatous polyposis, Glutamine synthetase, Beta-catenin, Immunohistochemistry

Glutamine synthetase (GS) has been established as a useful immunohistological adjunct in the diagnostic procedure of hepatocellular adenoma (HCA) harboring *CTNNB1* mutations, being more sensitive than b-catenin immunohistochemistry (IHC), to identify this subtype of HCA. Immunohistologically, *CTNNB1* mutations in exon 3 non-S45, or exon 3 S45, or exon 7/8 in b-catenin-mutated HCA (b-HCA) and b-catenin-mutated inflammatory type HCA (b-IHCA) show characteristic GS patterns [1]. B-IHCA has concurrent mutations in the *JAK/STAT* pathway which determine its inflammatory nature, but not its GS pattern. The various *CTNNB1* mutations lead to different GS expression patterns and different levels of activation of the b-catenin pathway which on its turn results in variable degrees of risk of malignant transformation [2]. In this issue of VIAR, Toth et al. presented 3 cases and reviewed 8 published cases of HCA in patients with familial adenomatous polyposis (FAP-HCA) [3]. Recently, another case of FAP-HCA has been reported [4].

Histological, immunohistological, and molecular data of the published cases were available in varying combinations and, when analyzed in more details, showed variable immunohistological and molecular profiles. Of the 12 published cases, 2 have been molecularly or immunohistologically confirmed as a HNF1a-HCA (H-HCA) and 3 as IHCA. One H-HCA case also contained hepatocellular carcinoma (HCC) adjacent to the HCA. In 2 cases, only germline but no somatic *APC* mutation was found, 1 case had no somatic APC mutation but there was loss of heterozygosity while in 3 cases there was biallelic *APC* mutation. GS IHC was done in 6 patients of whom 1 was negative and 5 diffusely positive. The GS-negative case and 3 of the 5 GS-positive patients had all negative nuclear b-catenin and absence of *CTNNB1* mutation. Additional data (*partly unpublished*) concern 2 personal FAP patients (1 included in the 12 reviewed cases), with diffuse GS HCA. A 26-year-old man with one HCA contains rare nuclear b-catenin-positive cells and mild cytological atypia. In the other case, a 37-year-old woman had multiple GS-positive HCA without nuclear b-catenin, but also a GS- and nuclear b-catenin-positive HCC without b-catenin mutation. The case of Kusnik et al. is another case showing nuclear b-catenin but there were no data of GS IHC and *CTNNB1* mutation [4].

Despite the limited number of patients, it is important to note that in specific contexts, diffuse GS expression in HCA does not automatically represent *CTNNB1* mutation. However, diffuse GS expression in FAP does imply b-catenin activation pathway, as GS is encoded by *GLUL*, a target gene upregulated by b-catenin. In FAP, *APC* mutation results in disruption of b-catenin degradation with subsequent b-catenin accumulation and activation [5]. Absence of nuclear b-catenin despite biallelic *APC* mutation can probably be explained by lower levels of b-catenin activation than when *CTNNB1* mutation is present. The loss of function due to APC mutation may not be complete, leaving some residual capacity of the protein, hence also of b-catenin degradation [5]. The presence of nuclear b-catenin also does not necessarily represent *CTNNB1* mutation, as has been shown in some FAP-associated hepatoblastoma [6] and in high-grade dysplastic intraductal papillary neoplasm of the bile duct (IPNB) with somatic *APC* mutation [7]. Similarly, the nuclear b-catenin in Kusnik’s FAP-HCA may also represent b-catenin activation due to *APC* mutation without *CTNNB1* mutation. The findings in these 3 studies signify the role of b-catenin in carcinogenesis in these contexts despite absence of *CTNNB1* mutation. The nuclear b-catenin in Kusnik’s case was accompanied by mild cytomorphological atypia and focal disruption of reticulin, rendering this case a borderline HCA [2], in contrast with Toth’s 3 cases which did not contain cytomorphological atypia combined with absence of nuclear b-catenin.

In short, although some FAP-HCA cases show a less consistent pattern regarding the association of GS and nuclear b-catenin expression compared to b-(I)HCA, GS- and/or nuclear b-catenin-positive FAP-HCA do contain b-catenin activation despite the absence of *CTNNB1* mutation. Toth et al. rightly pointed out that the GS-positive FAP-HCA cannot be readily classified as b-(I)HCA as there was no *CTNNB1* mutation, which is the criterion of b-(I)HCA. Nevertheless, it is a matter of debate, whether or not the FAP-related HCA should be classified as a distinct subgroup of HCA. The lack of a uniform histological, immunohistological, and molecular profile in the limited number of published cases so far do not offer a solid base for a separate categorization.

HCA has been documented in a variety of co-morbidities such as vascular diseases, glycogenosis, and tyrosinemia [8]. Two GS- and nuclear b-catenin-positive HCA in glycogenosis 1 have been reported, one of which had no *CTNNB1* mutation and the other one had no molecular data. [9]. Based on the above, awareness of an unusual GS and nuclear b-catenin expression in HCA in FAP patients is pivotal for the clinical practice. Also, that in special clinical contexts diffuse GS expression in HCA is not necessarily the result of *CTNNB1* mutation.

As shown by Toth et al. it is of paramount clinical importance to perform histology of focal hepatic lesions in FAP patients. Since 4/12 published FAP-HCA patients had multiple nodules, those could have been misinterpreted as metastatic colorectal carcinoma if histology was not performed. And in case an HCA is found, it is vital to document its cytomorphological, immunohistological, and molecular profile. Due to the rarity of HCA occurring in FAP patients the risk of malignancy is uncertain. However, as 3 young FAP patients out of the 12 published cases showed a borderline HCA in one case and a concurrent HCC in 2 cases, respectively, HCA in FAP patients seem to contain a non-negligible risk of malignant progression. Hence, a close clinical follow-up is justified.
